# Can the Cartilaginous Thickness Determine the Risk of Malignancy in Pelvic Cartilaginous Tumors, and How Accurate is the Preoperative Biopsy of These Tumors?

**DOI:** 10.1097/CORR.0000000000003065

**Published:** 2024-04-09

**Authors:** Minna K. Laitinen, Michael C. Parry, Guy V. Morris, Vineet Kurisunkal, Jonathan D. Stevenson, Lee M. Jeys

**Affiliations:** 1 Department of Orthopedics and Traumatology, Helsinki University Central Hospital, University of Helsinki, Helsinki, Finland; 2 Royal Orthopaedic Hospital, Birmingham, UK

## Abstract

**Background:**

Peripheral osteochondral tumors are common, and the management of tumors presenting in the pelvis is challenging and a controversial topic. Some have suggested that cartilage cap thickness may indicate malignant potential, but this supposition is not well validated.

**Questions/purposes:**

(1) How accurate is preoperative biopsy in determining whether a peripheral cartilage tumor of the pelvis is benign or malignant? (2) Is the thickness of the cartilage cap as determined by MRI associated with the likelihood that a given peripheral cartilage tumor is malignant? (3) What is local recurrence-free survival (LRFS), metastasis-free survival (MFS), and disease-specific survival (DSS) in peripheral chondrosarcoma of the pelvis and is it associated with surgical margin?

**Methods:**

Between 2005 and 2022, 289 patients had diagnoses of peripheral cartilage tumors of the pelvis (either pedunculated or sessile) and were treated at one tertiary sarcoma center (the Royal Orthopaedic Hospital, Birmingham, UK). These patients were identified retrospectively from a longitudinally maintained institutional database. Those whose tumors were asymptomatic and discovered incidentally and had cartilage caps ≤ 1.5 cm were discharged (95 patients), leaving 194 patients with tumors that were either symptomatic or had cartilage caps > 1.5 cm. Tumors that were asymptomatic and had a cartilage cap > 1.5 cm were followed with MRIs for 2 years and discharged without biopsy if the tumors did not grow or change in appearance (15 patients). Patients with symptomatic tumors that had cartilage caps ≤ 1.5 cm underwent removal without biopsy (63 patients). A total of 82 patients (63 with caps ≤ 1.5 cm and 19 with caps > 1.5 cm, whose treatment deviated from the routine at the time) had their tumors removed without biopsy. This left 97 patients who underwent biopsy before removal of peripheral cartilage tumors of the pelvis, and this was the group we used to answer research question 1. The thickness of the cartilage cap was recorded from MRI and measuring to the nearest millimeter, with measurements taken perpendicular in the plane that best allowed the greatest measurement. Patient survival rates were assessed using the Kaplan-Meier method with 95% confidence intervals as median observation times to estimate MFS, LRFS, and DSS.

**Results:**

Of malignant tumors biopsied, in 49% (40 of 82), the biopsy result was recorded as benign (or was considered uncertain regarding malignancy). A malignant diagnosis was correctly reported in biopsy reports in 51% (42 of 82) of patients, and if biopsy samples with uncertainty regarding malignancy were excluded, the biopsy identified a lesion as being malignant in 84% (42 of 50) of patients. The biopsy results correlated with the final histologic grade as recorded from the resected specimen in only 33% (27 of 82) of patients. Among these 82 patients, 15 biopsies underestimated the final histologic grade. The median cartilage cap thickness for all benign osteochondromas was 0.5 cm (range 0.1 to 4.0 cm), and the median cartilage cap thickness for malignant peripheral chondrosarcomas was 8.0 cm (range 3.0 to 19 cm, difference of medians 7.5 cm; p < 0.01). LRFS was 49% (95% CI 35% to 63%) at 3 years for patients with malignant peripheral tumors with < 1-mm margins, and LRFS was 97% (95% CI 92% to 100%) for patients with malignant peripheral tumors with ≥ 1-mm margins (p < 0.01). DSS was 100% at 3 years for Grade 1 chondrosarcomas, 94% (95% CI 86% to 100%) at 3 years for Grade 2 chondrosarcomas, 73% (95% CI 47% to 99%) at 3 and 5 years for Grade 3 chondrosarcomas, and 20% (95% CI 0% to 55%) at 3 and 5 years for dedifferentiated chondrosarcomas (p < 0.01). DSS was 87% (95% CI 78% to 96%) at 3 years for patients with malignant peripheral tumors with < 1-mm margin, and DSS was 100% at 3 years for patients with malignant peripheral tumors with ≥ 1-mm margins (p = 0.01).

**Conclusion:**

A thin cartilage cap (< 3 cm) is characteristic of benign osteochondroma. The likelihood of a cartilage tumor being malignant increases after the cartilage cap thickness exceeds 3 cm. In our experience, preoperative biopsy results were not reliably associated with the final histologic grade or malignancy, being accurate in only 33% of patients. We therefore recommend observation for 2 years for patients with pelvic osteochondromas in which the cap thickness is < 1.5 cm and there is no associated pain. For patients with tumors in which the cap thickness is 1.5 to 3 cm, we recommend either close observation for 2 years or resection, depending on the treating physician’s decision. We recommend excision in patients whose pelvic osteochondromas show an increase in thickness or pain, preferably before the cartilage cap thickness is 3 cm. We propose that surgical resection of peripheral cartilage tumors in which the cartilage cap exceeds 3 cm (aiming for clear margins) is reasonable without preoperative biopsy; the role of preoperative biopsy is less helpful because radiologic measurement of the cartilage cap thickness appears to be accurately associated with malignancy. Biopsy might be helpful in patients in whom there is diagnostic uncertainty or when confirming the necessity of extensive surgical procedures. Future studies should evaluate other preoperative tumor qualities in differentiating malignant peripheral cartilage tumors from benign tumors.

**Level of Evidence:**

Level III, diagnostic study.

## Introduction

Osteochondromas are common tumors of bone, constituting 20% to 50% of all benign bone tumors. Although osteochondromas are usually asymptomatic in the pelvis, they may cause pain through inflamed bursa or irritation of tendons, muscles, or skin or compressive symptoms on the urethra or bladder and thereby might benefit from surgery [8, 17]. It may be difficult to distinguish osteochondromas from peripheral chondrosarcomas in the pelvis, and a benign osteochondroma might transform over time into malignant peripheral chondrosarcoma. Therefore, it is most important to distinguish purely benign from malignant chondrosarcomas [4, 11, 14]. Transformation, when it occurs, is in the cartilaginous component of the lesion and appears to be related to the cartilage cap’s thickness. Continued growth after skeletal maturity and a hyaline cartilage cap greater than 1.5 cm in thickness in skeletally mature patients have been reported to suggest malignant transformation [18], but diverse widths of > 3 cm among pediatric patients and > 2 cm in adult populations have been reported [2, 13]. The diagnostic accuracy of preoperative biopsy has been shown to be between 36% and 86%, compared with the diagnosis after resection of any chondrosarcoma tumors [9, 12, 20] and is less investigated in peripheral chondrosarcomas.

There remains debate as to whether the cartilage cap thickness is a good marker for malignant potential [11], and if so, how thick a cartilage cap must be in order for it to be clinically concerning [16, 24]. Furthermore, there is little knowledge about the accuracy and role of preoperative biopsy in these tumors.

We therefore asked: (1) How accurate is preoperative biopsy in determining whether a peripheral cartilage tumor of the pelvis is benign or malignant? (2) Is the thickness of the cartilage cap as determined by MRI associated with the likelihood that a given peripheral cartilage tumor is malignant? (3) What is local recurrence-free survival (LRFS), metastasis-free survival (MFS), and disease-specific survival (DSS) in peripheral chondrosarcoma of the pelvis and is it associated with surgical margin?

## Patients and Methods

### Study Design and Setting

This was a retrospective study drawn from a longitudinally maintained database at a tertiary-care sarcoma center (the Royal Orthopaedic Hospital, Birmingham, UK).

### Patients

Between 2005 and 2022, 289 patients had diagnoses of peripheral cartilage tumors of the pelvis (either pedunculated or sessile) and were treated at the study center. Osteochondromas might appear either in a sessile or a pedunculated form; however, because the aim of this study was to investigate cartilage cap thickness in relation to malignancy, all osteochondromas are defined as peripheral cartilage tumors in this report (benign or malignant). Symptomatic osteochondromas were mostly located in the outer table of the ilium or in the pubic bone.

**Fig. 1:**
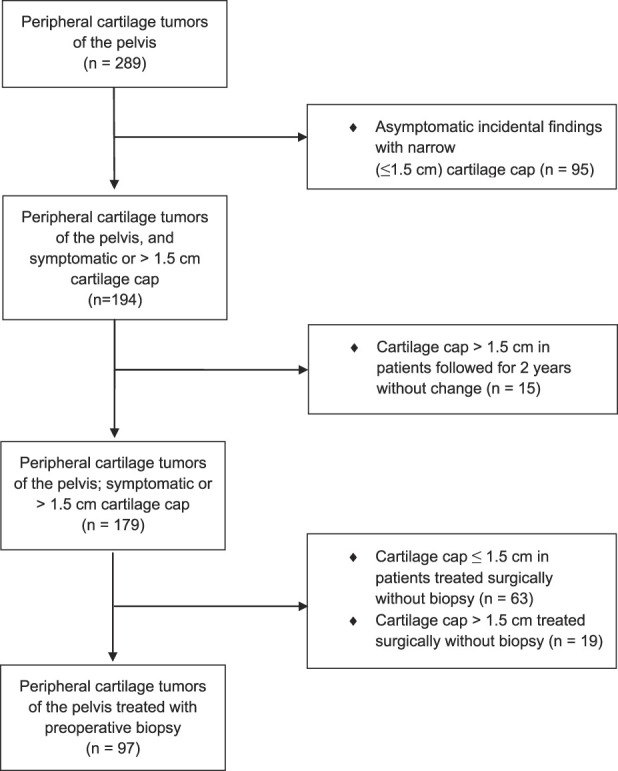
This flowchart represents the patient population.

Those whose tumors were asymptomatic and discovered incidentally and had cartilage caps ≤ 1.5 cm were discharged (95 patients), leaving 194 patients with tumors that were either symptomatic or had cartilage caps > 1.5 cm. Patients with tumors that were asymptomatic and had a cartilage cap > 1.5 cm were followed with MRIs for 2 years and were discharged without biopsy if the tumors did not grow or change in appearance (15 patients). Patients with symptomatic tumors that had cartilage caps ≤ 1.5 cm underwent removal without biopsy (63 patients). A total of 82 patients had their tumors removed without biopsy. Of those, 63 had caps ≤ 1.5 cm and 19 had caps > 1.5 cm; the latter group’s treatment deviated from the routine at the time. We were unable to ascertain why (presumably it pertained to the preference of the patient or the surgeon, or the recommendation of the tumor board), but given that this is a retrospective study and this group represented a relatively small subset of the original 194 patients, we did not feel it to be a disqualifying issue for our analysis. This left 97 patients who underwent biopsy before removal of peripheral cartilage tumors of the pelvis, and this was the group we used to answer our first research question about the accuracy of biopsy (Fig. 1). Biopsy was taken of 54% (97 to 179) of patients. Pathologic reports of surgical excision specimens and MRI were available for all patients. The biopsy specimen and resected tumor were evaluated by the same pathologists. Most of the benign tumors (81% [63 of 78]) did not have a preoperative biopsy, but when done, all biopsies showed no evidence of malignancy.

### Histology, Imaging, and Biopsy

Histologic grades were determined based on cellularity, nuclear size, the presence of an abundant hyaline cartilage matrix or mucomyxoid matrix, and atypical mitoses [6, 12]. When performed, the preoperative biopsy and resection histology reports were studied for tumor metrics. Thickness of the cartilage cap was recorded from MRI measuring to the nearest millimeter, with measurements taken perpendicular in the plane that best allowed the greatest measurement. MRI findings before surgery were available for all patients. The thickness of the cartilage cap was also counted from the pathology reports. We observed that the measurements on MRI and pathology specimens were within a few mm of each other.

All biopsies were performed at the referral center. Histologic grades of malignant lesions were determined based on cellularity, nuclear size, and the presence of an abundant hyaline cartilage matrix or mucomyxoid matrix and mitoses. Preoperative biopsy (when done) and resection histology were recorded from pathology reports. In patients with a malignant peripheral chondrosarcoma, 19% (19 of 101) did not have a preoperative biopsy. In 81% (82 of 101) of patients, a needle biopsy with imaging guidance (radiography or CT) was conducted. The preoperative biopsy results confirmed the presence of a cartilage tumor in all patients.

### Descriptive Data

Pathology review identified that 44% (78 of 179) of patients had benign osteochondroma and 56% (101 of 179) had malignant peripheral chondrosarcomas.

There was a slight male predominance of patients with benign and malignant tumors, with 69% (54 of 78) of patients being male in the benign group and 59% (60 of 101) in the malignant tumor group (Table 1). The ilium was the most commonly involved bone (63% [49 of 78] of patients with benign tumors and 60% [61 of 101] with malignant tumors) (Table 1). For malignant tumors, the histologic grade was Grade 1 in 42% (42 of 101) of patients, Grade 2 in 42% (42 of 101), Grade 3 in 11% (11 of 101), and dedifferentiated in 6% (6 of 101). In patients with benign tumors, 17% (13 of 78) had an underlying diagnosis of hereditary multiple exostosis, and in patients with malignant tumors, 24% (24 of 101) had hereditary multiple exostosis as an underlying diagnosis. Tumors were Grade 1 in 8 of these 24 patients, Grade 2 in 7, Grade 3 in 2, and dedifferentiated in 2 patients.

**Table 1.** Demographics

Patient characteristic		Benign	Malignant	p value
Total number of patients		44% (78)	56% (101)	
Age in years		29 (23-41)	40 (31-55)	< 0.001
Male		69% (54)	59% (60)	0.12
Tumor size in cm		6.0 (4.2-8.0)	12 (9.8-16)	< 0.01
Cartilage cap size in cm		0.5 (0.3-1.5)	8.0 (5.5-10)	< 0.01
Type of background	MHE	17% (13)	24% (24)	0.35
	Solitary	83% (65)	76% (77)	
Site	Ilium	63% (49)	60% (61)	0.14
	Acetabulum	4% (3)	3% (3)	
	Pubis	28% (22)	22% (22)	
	Ischiadicum	3% (2)	9% (9)	
	Sacrum	3% (2)	1% (1)	
	Hemipelvis^a^	-	5% (5)	
Biopsy before surgery		19% (15)	91% (91)	< 0.01
Histologic grade	Grade 1		42% (42)	< 0.01
	Grade 2		42% (42)	
	Grade 3		11% (11)	
	Dedifferentiated		6% (6)	
Metastasis		0	10% (11)	< 0.01
Median time to metastasis in months		0	15 (5-50)	< 0.01
Local recurrence		0	31% (31)	< 0.01
Median time to local recurrence in months		0	32 (15-41)	< 0.01
Status at last follow-up	NED	100% (78)	78% (79)	< 0.01
	AWD	-	3% (3)	
	DOD	-	15% (15)	
	DOO	-	4% (4)	
Follow-up in months		6 (1-41)	65 (30-124)	< 0.01

Data are presented as % (n) or median (IQR).

a Hemipelvis means tumor that was so large that the original site of the pedunculated tumor was not possible to define. All of these patients had MHE and there were multiple pedunculated sites, and due to the large cartilage tumor, it was not possible to know which was the primary site. MHE = multiple hereditary exostosis; NED = no evidence of disease; AWD = alive with disease; DOD = dead of disease; DOO = dead of other reasons.

The median duration of postoperative follow-up for benign osteochondromas was 6 months (range 0 to 222 months), and for peripheral chondrosarcomas, it was 65 months (range 0 to 270 months). Of those resected, 31% (56 of 179) of patients were lost to follow-up before 2 years without meeting a study endpoint (local recurrence, metastasis, or death), and another 3% (6 of 179) died because of their chondrosarcomas before 2 years, leaving 67% (120 of 179) of the original group who had either follow-up of at least 2 years or who had met a study endpoint before that minimum surveillance duration. Another 13% (24 of 179) had a minimum of 2 years follow-up but were not seen in the last 5 years; these were included in the survivorship analysis, but we cannot be sure of their status.

### Surgical Margins

The histologic status of the surgical margin was defined as microscopically positive (0 mm), negative (< 1 mm), or negative (≥ 1 mm). After surgery, patients with benign osteochondroma were followed for surgical healing, but they were advised to be in contact if there was concern of reoccurring tumor. None of the patients made any further contact. Patients with malignant tumors were followed according to European Sarcoma Network Working Group guidelines [9].

### Statistical Analysis

Patient survival rates were assessed using the Kaplan-Meier method with 95% confidence intervals as median observation times to estimate MFS, LRFS, and DSS. DSS was defined as the time from diagnosis to disease-related death and was censored at the date of the latest follow-up examination or death owing to other causes. LRFS and MFS were defined as the time from the surgical procedure to local recurrence (LR) or radiologic diagnosis of metastasis, respectively, and were censored at the date of the latest follow-up visit or death. LR was defined as tumor relapse evidenced by radiologic confirmation and subsequent histologic confirmation from biopsy, or by interval increase in the size of abnormal lesions on sequential imaging. Continuous variables are reported as median and range, and between-group differences were analyzed using the one-way Mann-Whitney test. The Pearson chi-square test was used to compare variables between groups, and the Mann-Whitney U-test was used for medians between groups. Univariate analysis was performed by comparing groups using a log-rank test, with subsequent univariate and multivariate Cox proportional hazard analyses of continuous variables to identify factors associated with LRFS and DSS. The subdistribution hazard ratio of the role of LR on survival was calculated using a competing risk analysis. Synchronous metastases (metastases that developed before LR, at the time of LR, or within 90 days after LR) and death for other reasons were considered competing events in analyses of the role of LR on DSS. Receiver operating characteristic curve analysis was performed to evaluate specificity and sensitivity and to determine a cutoff point for the size of the cartilage cap between benign and malignant tumors. Statistical analyses were performed using SPSS Statistics 27.0 (IBM Corp) and STATA 17 (Stata). A p value < 0.05 was considered significant.

## Results

### Accuracy of Preoperative Biopsy

Of malignant tumors biopsied, in 49% (40 of 82), the biopsy demonstrated a benign tumor in 8 patients or was uncertain regarding malignancy in 32. Malignancy was confirmed in 51% (42 of 82) of patients who had a preresection biopsy. Of 50 patients with malignant tumors and a diagnostic biopsy, 84% (42 of 50) of tumors were correctly called malignant and 16% (8 of 50) were incorrectly diagnosed as benign osteochondromas. The biopsy results were concordant with the final histologic grade compared with the resection histology in only 33% (27 of 82) of patients. The grade based on biopsy compared with the final pathology was accurate in 54% (13 of 24) of Grade 1 lesions, 77% (10 of 13) of Grade 2 lesions, and 100% (5 of 5) of Grade 3 and undifferentiated lesions. Among these 82 patients, 15 biopsies underestimated the final histologic grade (Table 2).

**Table 2.** Concordance of histologic grade between biopsy and resection specimens

Biopsy result	Surgical specimen			
	Grade 1	Grade 2	Grade 3	Dedifferentiated
No biopsy (n = 19)	9	5	3	2
No evidence of malignancy (n = 8)	6	2	0	0
Uncertain regarding malignancy (n = 32)	14	16	2	0
Grade 1 (n = 24)	13	9	2	0
Grade 2 (n = 13)	0	10	2	1
Grade 3 (n = 3)	0	0	2	1
Dedifferentiated (n = 2)	0	0	0	2
Total (n = 101)	42	42	11	6

### Cartilage Cap Thickness

**Fig. 2:**
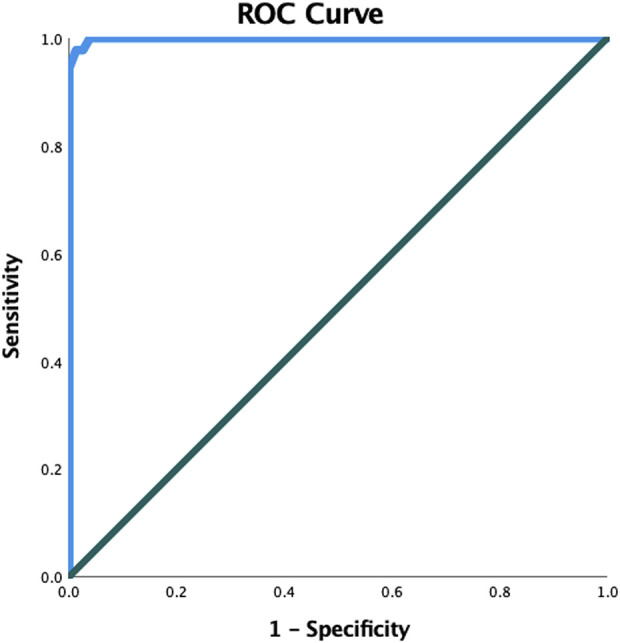
We used receiver operator characteristic curves to find cartilage cap cutoff values to differentiate between benign and malignant tumors. The cartilage cap cutoff value associated with malignancy was 2.9 cm (sensitivity 96%, specificity 100%).

Tumors with cartilage caps 3 cm or larger were much more likely to be malignant, and using 3 cm as the cutoff yielded a sensitivity of 96% and specificity of 100% (Fig. 2).

**Fig. 3:**
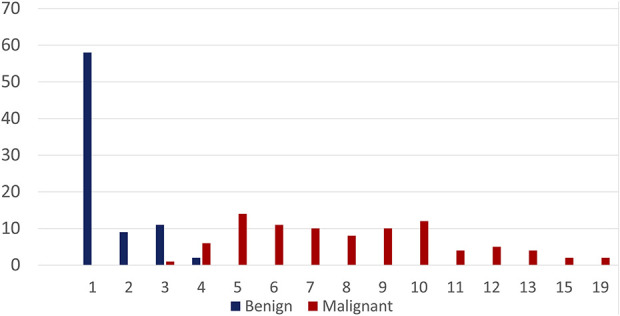
This graph shows the size of the cartilage gap in cm, stratified by benign versus malignant histology. A color image accompanies the online version of this article.

**Fig. 4:**
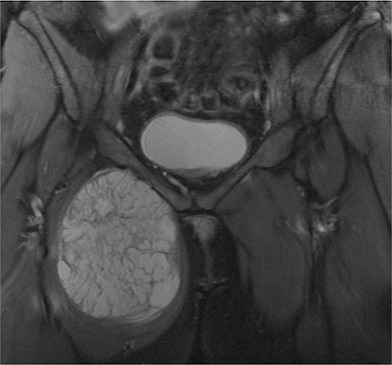
A 48-year-old woman had a Grade 2 peripheral chondrosarcoma of the right pubic bone. MRI shows a 13-cm-thick cartilage cap.

The median cartilage cap thickness for all benign osteochondromas was 0.5 cm (range 0.1 to 4.0 cm). The median cartilage cap thickness for malignant peripheral chondrosarcomas was 8.0 cm (range 3.0 to 19 cm, difference of medians 7.5 cm; p < 0.01) (Fig. 3). The median cartilage cap thickness for Grade 1 malignant peripheral chondrosarcomas was 7.0 cm (range 3.5 to 19 cm), for Grade 2 malignant peripheral chondrosarcomas was 7.0 cm (range 3.0 to 19 cm), for Grade 3 malignant peripheral chondrosarcomas was 10 cm (range 5.0 to 15 cm), and for dedifferentiated malignant peripheral chondrosarcomas was 10 cm (range 5.0 to 15 cm) (Fig. 4). With the numbers we had, the cap thickness did not differ between the pathologic grades of the tumors because the thickness of the cartilage cap had similar median values between different grades.

### Survivorship

No patients with benign osteochondromas developed LR or died of disease. Thirty-one percent (31 of 101) of patients with malignant peripheral chondrosarcomas developed LR. Twenty-six percent (11 of 42) who developed LR were in patients with a resected Grade 1 tumor, 31% (13 of 42) were Grade 2, 45% (5 of 11) were Grade 3, and 33% (2 of 6) were dedifferentiated. Eleven patients with malignant peripheral chondrosarcomas developed metastases and all of them died of disease within a median of 15 months (range 0 of 221 months). Twelve percent of metastases were in patients with a resected Grade 2 tumor (5 of 42), 36% (4 of 11) were in Grade 3 tumors, and 33% (2 of 6) were dedifferentiated. Fifteen patients died of the disease, four of them after treatment. Additionally, among the cohort, three are alive with disease, 78 are alive without disease, and four died because of another disease.

**Fig. 5:**
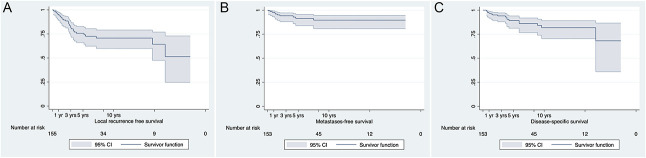
These graphs show (**A**) local recurrence-free survival, (**B**) metastases-free survival, and (**C**) disease-specific survival in patients with malignant peripheral chondrosarcomas at 1, 3, and 5 years.

**Fig. 6:**
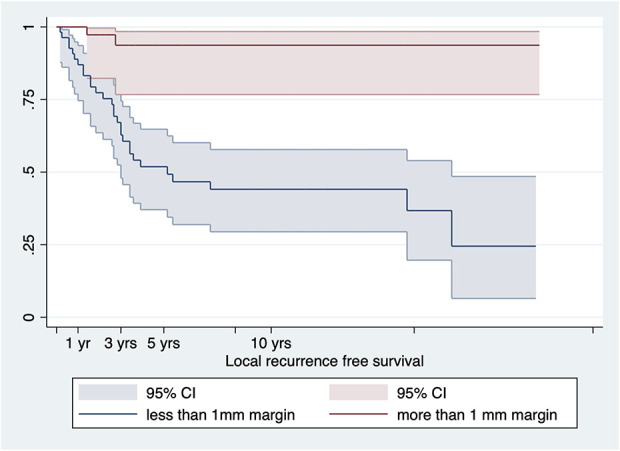
This graph shows local recurrence-free survival in patients with malignant peripheral chondrosarcoma stratified by a margin of less than 1 mm and 1 mm or more. A color image accompanies the online version of this article.

LRFS was 93% (95% CI 87% to 98%) at 1 year, 75% (95% CI 66% to 85%) at 3 years, and 68% (95% CI 58% to 78%) at 5 years for patients with malignant peripheral tumors (Fig. 5A). MFS was 96% (95% CI 91% to 100%) at 1 year, 86% (95% CI 77% to 95%) at 3 years, and 83% (95% CI 74% to 93%) at 5 years (Fig. 5B). DSS in patients with malignant peripheral chondrosarcoma was 96% (95% CI 92% to 100%) at 1 year, 92% (95% CI 87% to 98%) at 3 years, and 86% (95% CI 79% to 94%) at 5 years. (Fig. 5C). LRFS was 87% (95% CI 78% to 96%) at 1 year, 49% (95% CI 35% to 63%) at 3 years, and 25% (95% CI 2% to 48%) at 5 years for patients with malignant peripheral tumors with < 1-mm margins. LRFS was 100% at 1 year, 97% (95% CI 92% to 100%) at 3 years, and 94% (95% CI 85% to 100%) for patients with malignant peripheral tumors with ≥ 1-mm margin (p < 0.01) (Fig. 6). We found no association among tumor size, increasing thickness of the cartilage cap, and LRFS (HR 1.006 [95% CI 0.922 to 1.098]; p = 0.90 and HR 0.996 [95% CI 0.879 to 1.129]; p = 0.95, respectively) with the numbers available.

DSS was 100% at 1 and 3 years and 97% (95% CI 91% to 100%) at 5 years for Grade 1 chondrosarcomas; 100% at 1 year, 94% (95% CI 86% to 100%) at 3 years, and 90% (95% CI 79% to 100%) at 5 years for Grade 2 chondrosarcomas; 91% (95% CI 74% to 100%) at 1 year and 73% (95% CI 47% to 99%) at 3 and 5 years for Grade 3 chondrosarcomas; and 60% (95% CI 17% to 100%) at 1 year and 20% (95% CI 0% to 55%) at 3 and 5 years for dedifferentiated chondrosarcomas (p < 0.01). DSS was 93% (95% CI 86% to 100%) at 1 year, 87% (95% CI 78% to 96%) at 3 years, and 81% (95% CI 70% to 92%) at 5 years for patients with malignant peripheral tumors with < 1-mm margins. DDS was 100% at 1 and 3 years and 96% (95% CI 88% to 100%) at 5 years for patients with malignant peripheral tumors with ≥ 1-mm margins (p = 0.01). We found no association among tumor size, increasing thickness of the cartilage cap, and DSS (HR 10.48 [95% CI 0.950 to 1.155]; p = 0.35 and HR 1.109 [95% CI 0.971 to 1.266]; p = 0.13, respectively). We found an association between LR and poorer survival (subdistribution HR 6.37 [95% CI 1.81 to 22.35]; p < 0.01). None of the factors studied in the multivariate Cox regression analysis were associated with poorer DSS, with the numbers available (Table 3).

**Table 3.** Factors associated with disease-specific survival identified by multivariate analysis

Factor	Hazard ratio (95% CI)	p value
Margin more than 1 mm	0.05 (0.03-0.8)	0.04
Metastasis	7.37 (1.8-30)	0.01
Grade	3.76 (1.6-9)	0.01

## Discussion

Most osteochondromas are asymptomatic, but pelvic sarcomas may cause symptoms because of compression, fracture, bursitis, or increased size [25]. They grow slowly during childhood, with growth proportional to the overall immature skeleton, but should not enlarge after puberty [10]. A change in size postpuberty, often accompanied by new or worsening symptoms, suggests malignant transformation, occurring as an increase in the cartilage cap thickness leading to peripheral chondrosarcomas [15, 19]. Preoperative differentiation between benign and malignant peripheral cartilage tumors is challenging, however, impacting surgical treatment greatly. The importance of cartilage cap thickness in malignant transformation is recognized, but its reliability as a marker and clinical concern regarding thickness have remained uncertain.

In this study, we observed that the thickness of the cartilaginous cap in a peripheral cartilage tumor of the pelvis was associated with the likelihood of malignancy, and in our experience, preoperative biopsies were often nondiagnostic or inaccurate compared with final pathology reports.

### Limitations

First, the number of patients fulfilling the criteria for inclusion was small. Our primary concern, however, was to focus on the cutoff point of cartilage cap thickness where the incidence of malignancy increased. Because a cartilage cap showing benign histology and a cap showing malignant histology overlapped, a larger sample could provide a more accurate estimation of this cutoff point. Future analyses would benefit from including more patients, and having proposed this finding, the next step is for other high-volume centers to collaborate or refute this observation.

Second, the study was subject to selection bias. During the study period, there were general indications guiding biopsy choices. However, as in a retrospective study, we acknowledge that 10% (19 of 194) of surgical resections proceeded without preoperative biopsy, based on the index of suspicion of malignancy, which at the time did not fit the treatment plan we normally followed. These patients invariably displayed radiologic features of malignancy (large tumors with a thick cartilage cap), and their tumors were treated as malignant. As a potential flaw, this means some patients may have been overtreated, but invariably these patients presented with symptoms that warranted tumor excision, and the nature of that excision was governed by the radiologic features suggestive of malignancy.

Although we have demonstrated an association between the thickness of the cartilage cap and malignant potential of the tumor, there were no independent blind comparisons of diagnostic tests; therefore, these findings might overestimate our results and these findings need to be confirmed by larger studies from multiple centers [6, 23]. However, all specimens were assessed by specialist bone pathologists in a single center, which we believe will reduce this potential variability. Finally, there was a difference in the duration of follow-up between patients with benign tumors and those with malignant tumors. However, because all bone tumor service and surgery in our country is well-centralized, it is unlikely that any patients who developed recurrence would have been treated away from this center.

### Accuracy of Preoperative Biopsy

Our results are aligned with the findings of others in that, where performed for malignant lesions, biopsy results correctly agreed with the final histologic grade in only 33% of patients’ tumors [13, 22, 26]. Moreover, the biopsy result agreed with the final pathology report of malignancy in only 51% of patients. When considering the management of osteochondromas of the pelvis, assessing malignancy is of great importance, because inadvertent intralesional or resection with an inadequate margin of a presumed benign lesion that is subsequently confirmed as a malignant lesion is associated with a high risk of local recurrence and decreased DSS [3, 5, 26]. Our results have again highlighted that preresection biopsy of cartilaginous tumors, particularly around the pelvis, does not add to the diagnostic pathway; therefore, we feel biopsy is not mandatory before proceeding to surgical management of peripheral cartilage lesions of the pelvis. Biopsies might be helpful when diagnoses are uncertain or to validate the necessity of major surgeries. Decisions regarding the margin width at surgical resection should not be related to the grade demonstrated on biopsy, because this correlated with the resection histology grade in only 33% of patients, which may falsely reassure a treating surgeon.

### Cartilage Cap Thickness

The results from our study showed that a small cartilage cap thickness (< 3 cm) was characteristic of a benign osteochondroma, because no tumors with a cap thickness less than 3 cm were confirmed as malignant on resection histology. However, the likelihood of a patient having a malignant peripheral cartilage tumor increased when the cartilage cap exceeded 3 cm. The size of the cartilage cap did not differ between different grades of malignancy. Because all malignant tumors had a cartilage cap of 3 cm or more, we would recommend excision for pelvic osteochondromas, preferably before the cartilage cap reaches 3 cm on MRI. The ability to estimate cartilage cap thickness from MRI was strong, with the final pathology report indicating a difference from MRI estimation of only a few millimeters at most. The role of cartilage cap thickness as a marker of malignant transformation of osteochondroma to peripheral chondrosarcoma is recognized but remains open to debate. Although the thickness of the cartilage cap corresponds to an increased risk of a peripheral osteochondroma being malignant [1, 2, 6, 16], there is no guidance from other reports as to the appropriate cartilage cap size, but rather a wide spectrum of measurements. Therefore, it has been contended that the size of the cartilage cap could not in itself be used as an absolute criterion for malignant transformation. Some authors have stressed the importance of the character and quality of the cartilage cap in addition to its absolute size. Ahmed et al. [1] stated that a qualitative assessment of the cartilage cap was more helpful than precise measurement of cap thickness. In previous studies, MRI has been shown to allow accurate assessment, give good delineation of cartilage cap thickness and the extent of the tumor, and provides an excellent assessment of surrounding structures [3, 7, 24]. Where the thickness of the cartilage cap measures less than 3 cm and patients do not wish to undergo surgery, we recommend serial imaging of the lesion using MRI at regular intervals to assess for an increase in size of the cartilage cap and to guide the indication for subsequent excision.

### Survivorship

We have demonstrated no local recurrences after resection of a benign osteochondroma, aligning with reports of others demonstrating the effectiveness of excision in curing benign peripheral cartilage tumors. The existing studies do, however, report a very low rate of local recurrence, primarily observed in children or patients with hereditary multiple exostosis [3]. Notably, most chondrosarcoma research fails to distinguish between central and peripheral chondrosarcomas, despite differing clinical behaviors, particularly among higher-grade tumors. Our study demonstrates that none of the patients with low-grade (Grade 1) tumors developed metastases or died as a result of the disease, consistent with the current understanding, which is that of a generally low malignant potential for central and peripheral low-grade chondrosarcoma [13, 25, 27, 28]. Furthermore, our results emphasize that achieving a clear surgical resection margin, exceeding 1 mm, results in a very low local recurrence rate and high LRFS. LR was associated with decreased survival in our study.

### Conclusion

Preoperative biopsy findings were not reliably associated with the final histologic grade or malignancy, being accurate in showing the correct grade of peripheral chondrosarcomas in 33% and diagnosing malignancy in 50% of patients who had a diagnostic biopsy. A small cartilage cap (< 3 cm) was characteristic of a benign osteochondroma. The likelihood of a given peripheral cartilage tumor being malignant appears to increase after the cartilage cap exceeds 3 cm. We therefore would recommend observation for 2 years for patients with pelvic osteochondromas where the cap thickness is < 1.5 cm and there is no associated pain. If a patient’s tumor shows an increase in thickness or there is pain from pelvic osteochondromas, we recommend excision. In general, we recommend close observation or excision of pelvic peripheral cartilage tumors with cartilage caps > 1.5 cm and preferably before the cartilage cap thickness exceeds 3 cm. We feel that preoperative biopsy is seldom needed because radiologic measurement of the cartilage cap thickness was shown to be associated with a malignant tumor in our study, but confirmation of our biopsy findings at other centers and with more patients is needed. We propose that surgical resection of peripheral cartilage tumors where the cartilage cap exceeds 3 cm, aiming for clear margins, is reasonable treatment for malignant tumors without preoperative biopsy. Biopsy might be helpful in situations of diagnostic uncertainty or when confirming the necessity of extensive surgical procedures. An inconclusive biopsy or interpretation of the grade on biopsy should be treated with caution. and the histologic finding from biopsy should be correlated with radiologic findings and discussed by a multidisciplinary team before planning surgical treatment.
